# An Airborne Arc Array Synthetic Aperture Radar Vibration Error Compensation Method

**DOI:** 10.3390/s24031013

**Published:** 2024-02-04

**Authors:** Mengxue Xiao, Pingping Huang, Wei Xu, Weixian Tan, Zhiqi Gao, Yaolong Qi

**Affiliations:** 1 College of Information Engineering, Inner Mongolia University of Technology, Hohhot 010080, China; 20211100105@imut.edu.cn (M.X.); xuwei1983@imut.edu.cn (W.X.); wxtan@imut.edu.cn (W.T.); gzqngd@imut.edu.cn (Z.G.); qiyaolong@imut.edu.cn (Y.Q.); 2 Inner Mongolia Key Laboratory of Radar Technology and Application, Hohhot 010051, China

**Keywords:** arc array synthetic aperture radar, paired echoes, vibration compensation

## Abstract

Compared to conventional radars, arc array synthetic aperture radar (SAR) enables wide-area observation under ideal conditions. However, helicopters carrying arc array SAR platforms are generally smaller in size and more sensitive to vibration, which has a greater impact on the imaging quality. In this paper, the vibration error of the arc array SAR platform is investigated, and a vibration error model of the arc array SAR platform is established. Based on the study of the vibration error model, a vibration phase estimation and compensation algorithm based on the delayed conjugate multiplication method is proposed. In the first step, distance pulse pressure processing is performed on the echo signal. In the second step, the pulse pressure signals and their delays in the same distance unit are subjected to conjugate multiplication, and the phase of the signal after conjugate multiplication is extracted. The extracted phase is then amplitude- and phase-compensated to estimate the vibration phase. In the third step, the vibration phase is compensated in the azimuthal direction of the distance pulse pressure signal, and the pairwise echo is eliminated, which completes the compensation of the airborne arc array SAR vibration platform.

## 1. Introduction

SAR, as a microwave detection technology, has great advantages in the application of complex low-altitude conditions as well as harsh weather environments. It does not depend on the climatic environment, and it can carry out all-day and all-weather detection, regardless of whether it is day and night, and it is capable of high-resolution imaging of the observed scene, while the arc array SAR can be suspended in the air with a helicopter as the airborne platform, and the arc array unit can be used to scan the whole scene over a wide range of areas, improving the perception of the whole scene [1]. The new airborne arc array SAR imaging system consists of a microwave imaging device based on the arc array antenna, addressing the limitations of conventional SAR and linear array SAR observation regarding the use of a single viewpoint. Through the wave propagation direction of transmitter bandwidth signals, it establishes the distance to the resolution of the angle along the arc of the angle of the layout of multiple antenna array elements to form an arc-integrated aperture in order to achieve the column direction resolution. The imaging system can realize the real-time, dynamic, omni-directional, large-field-of-view 360° two-dimensional imaging observation of the environment around the platform, which can provide a guarantee for the flight safety of helicopters [2]. In practice, the rotor and engine of the arc array platform often cause the platform to vibrate, which impacts the instantaneous distance model between the radar platform and the target, and ultimately leads to defocused imaging, which seriously affects the image resolution [3].

Due to the periodic time-varying nature of airborne platform vibration, it is often difficult to accurately compensate for the vibration error via analytical equations when the vibration parameters are unknown. This is due to the complex nonlinear Doppler phase modulation of the SAR echo by the vibrating target, which results in a broadening of the echo spectrum, the so-called micro-Doppler effect [4]. The micro-Doppler effect destroys the characteristics of the echo signal on which conventional SAR imaging algorithms rely. In addition, the high-frequency vibration of the platform generates a large number of paired echoes in the azimuthal direction, which leads to attenuation of the main image’s intensity and image scattering [5]. 

The modelling of a vibrating target mainly adopts the scattering point model, which usually provides the corresponding distance equations according to the vibration types and geometries of different targets, derives the echo model according to the working principle of synthetic aperture radar [6,7], performs parameter estimation and feature extraction of the echo model [8], and compensates for it by using the relevant algorithms to obtain the focused image. Micro-Doppler feature extraction and parameter estimation are important issues in vibrating target detection of synthetic aperture radar (SAR), which can mainly be classified into three types of methods based on the image domain, time-frequency analysis and transform domain. Image-domain-based micro-Doppler feature extraction and parameter estimation methods mainly take advantage of the high-resolution capability of synthetic aperture radar to extract the radial micromotion history of the scattering center of the vibrating target with a one-dimensional high-resolution distance-directed sequence [9]. Time-frequency analysis is mainly divided into linear time-frequency analysis, bilinear time-frequency analysis and high-order time-frequency analysis. Linear time-frequency analysis has the advantages of a small arithmetic volume and no cross terms, but the focusing performance is poor [10]. Bilinear time-frequency analysis has good time-frequency focusing performance, but there are cross terms. Higher-order time-frequency analysis mainly consists of a Fourier transform of the local warning autocorrelation function of the signal, which has good time-frequency aggregation for higher-order phase signals and is suitable for analyzing signals with faster frequency changes, but is more sensitive to noise [11]. Transform domain-based micro-Doppler feature extraction and parameter estimation methods are often combined with time-frequency analysis methods, which are mainly used to perform time-frequency analysis on the original azimuthal signals and improve the distribution structure of the micro-Doppler signals in the original domain by seeking a variety of transform domains, so as to better extract the signal features [12].

In addition, the problem of compensating for the periodic vibration of SAR imaging platforms has also been a major hotspot of research in recent years. Generally speaking, SAR imaging can compensate for the echo data by combining the platform operation information recorded by the inertial navigation system (INS) and global positioning system (GPS) on the radar platform. However, there are still residual phase errors in the echo signals after INS and GPS compensation, and high-precision imaging cannot be achieved. Since the mid-1970s, the SAR imaging sub-focusing technique has been important in improving the quality of radar imaging. Depending on whether the processing requires the presence of isolated strong scattering points in the scene, the self-focusing algorithms can usually be classified into two categories. The first category requires the presence of isolated strong scattering points in the scene, including the Map Drift (MD) algorithm [13], the Multiple Aperture Map Drift (MAM) [14] algorithm, the Phase Gradient Autofocus (PGA) algorithm [15,16], etc. The second category does not require the presence of isolated strong scattering points in the scene, and is based on the image quality of the autofocus methods, including Phase Adjustment by Contrast Enhancement (PACE) algorithm [17,18], the Minimum Entropy Autofocus (MEA) [19,20] algorithm, the Maximum Image Contrast Autofocus (MICA) [21] algorithm based on coordinate descent optimization, and so on. 

In recent years, scholars at home and abroad have actively explored SAR vibration compensation technology, and they have completed a lot of basic work on radar micro-motion feature extraction, along with studying the method of detecting micro-motion targets by using time-frequency analysis or spectrum analysis and estimating the micro-motion parameters by means of wavelet and independent component analysis. In addition, relevant scholars have made use of the inverse Radon transform and Hough transform, which have good focusing performance on sinusoidal curves, to carry out the inverse Radon transform [22] on the time-frequency map of micromotion target echoes, being able to achieve the detection of micromotion targets and the estimation of parameters against a noisy background to achieve the estimation of and compensation for the vibration parameters of SAR. In this paper, based on the arc array SAR, we study the impact of vibration on imaging and propose an effective vibration compensation method.

This paper is organized as follows. Section 2 presents the imaging model and the simplified vibration geometry configuration of the arc array SAR, while the geometry-based echo signal model is established. Section 3 derives the effect of the vibration error phase on imaging quality based on the vibration error model of the arc array SAR platform. A vibration phase estimation and compensation algorithm based on delayed conjugate phase multiplication is proposed in Section 4. The vibration effects and the vibration compensation algorithm are simulated and analyzed, respectively, in Section 5 to verify the correctness of the theoretical derivation and the effectiveness of the compensation algorithm. Conclusions are given in Section 6.

## 2. Arc Array Imaging Geometry

### 2.1. Instantaneous Slant Distance Model

**Figure 1:**
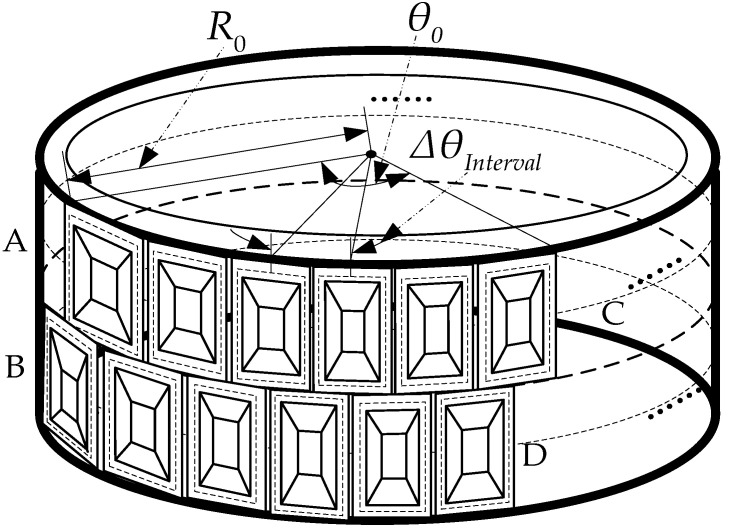
Arc array configuration diagram.

As shown in Figure 1, an arc array sends and receives through its antennas a plurality of array elements along the arc in the form of equal spacing, equal angle, and equal height to be placed, and through the microwave switch achieves signal transmission and reception. The transmitter and receiver antennas are arranged in relation to the arc at a certain angle, pointing to the outside of the center of the circle, thus forming a synthetic aperture of the arc, in order to achieve a high resolution for array. The angular spacing between adjacent antenna elements is set to be the same $\Delta \theta_{Interval}$, with a radius of $R_{0}$. $\;\theta_{0}$ denotes the synthetic aperture angle of a curved array antenna, with A and C being the transmitting array elements, and B and D being the receiving array elements.

The arc array SAR adopts frequency-modulated continuous wave (FMCW) operation, and its transmitted signal expression is

(1) $$s_{tr}\left(t\right)=\mathrm{rect}\left(\frac{t}{T_{r}}\right)\exp\left[j2\pi \left(f_{c}+\frac{1}{2}K_{r}t^{2}\right)\right]$$

where *f_c_* is the center frequency of the radar, *t* is the distance to fast time variable, *T_r_* is the duration of the signal emission on a single array element, *K_r_* is the linear modulation frequency of the signal, and the signal bandwidth is $B_{r}=K_{r}T_{r}$.

**Figure 2:**
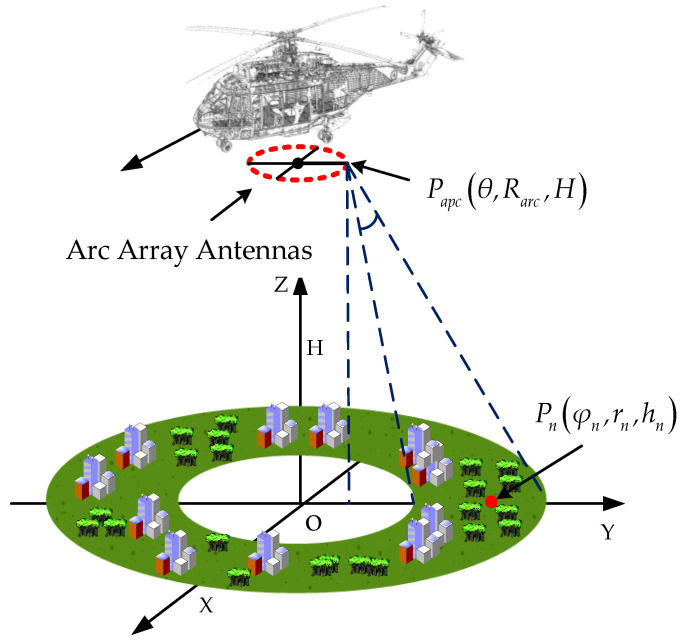
Arc array imaging model.

The real imaging scene of the arc array SAR is shown in Figure 2, where the red dashed circle stands for arc array antennas. the equivalent sampling point is $P_{apc}\left(\theta ,R_{arc},H\right)$, the target point is $P_{n}\left(\varphi_{n},r_{n},h_{n}\right)$, the angle between the Y-axis and the equivalent sampling point is θ, and the angle between the Y-axis and the target point is $\varphi_{n}$. Ideally, the instantaneous distance between the equivalent sampling point and the target point can be expressed as

(2) $$R=\sqrt{{\left(R_{arc}\cos\theta -r_{n}\cos\varphi_{n}\right)}^{2}+{\left(R_{arc}\sin\theta -r_{n}\sin\varphi_{n}\right)}^{2}+{\left(H-h_{n}\right)}^{2}}$$

The equivalent sampling point $P_{apc}$ on the curved array antenna receives the echo signal scattered by the point target $P_{n}$ in the scene as follows:

(3) $$\begin{array}{ll} ss_{re}\left(t,\theta \right)= & \sigma \left(\theta_{n},r_{n},h_{n}\right)\mathrm{rect}\left(\frac{t-R\left(\theta \right)/c}{T_{r}}\right)\mathrm{rect}\left(\frac{\theta -\varphi_{n}}{\theta_{a}}\right)\\ & \cdot \exp\left\{j2\pi \left[f_{c}\left(t-2R\left(\theta \right)/c\right)+\frac{1}{2}K_{r}{\left(t-\frac{2R\left(\theta \right)}{c}\right)}^{2}\right]\right\} \end{array}$$

where $\sigma (\theta_{n},r_{n},h_{n})$ represents the scattering coefficient, *R*(*θ*) represents the instantaneous slant distance, and $\theta_{a}$ represents the beamwidth of the antenna elements in the horizontal direction, i.e., the array-wise beamwidth.

### 2.2. Resolution Analysis

The resolution in the distance direction is achieved by transmitting the bandwidth signal through the arc array antenna, and its resolution is determined only by the bandwidth of the transmitted signal; the aperture of the arc is formed by the antenna array elements arranged along the arc angle, and then the equivalent phase center processing is carried out, so as to achieve a high resolution in the orientation direction via the principle of synthetic aperture. The resolution of the arc array SAR in the distance and azimuth directions is mainly determined by the bandwidth of the transmitted signal, the size of the radius of the arc array and the beamwidth of the antenna array elements in the array direction. The system transmits and receives FMCW signals, so the effective signal bandwidth in the distance upward is related to the distance between the target and the antenna unit, and the effective signal bandwidth B is calculated as follows:

(4) $$B=B_{r}\left(1-\frac{\tau_{n}}{T_{r}}\right)$$

The geotropic resolution $\rho_{r}$ of the arc array SAR imaging system can be calculated as

(5) $$\rho_{r}=\frac{c}{2B\cos\beta_{n}}=\frac{c}{2B_{r}(1-\tau_{n}/T_{r})\cos\beta_{n}}$$

where $\tau_{n}$ is the delay of the point target echo in the imaging scene, *B_r_* is the bandwidth of the transmitted FMCW signal, $T_{r}$ is the time that a single antenna array element continues to transmit the signal, and $\beta_{n}$ is the angle between the line of sight of the point target observation and the ground.

The magnitude of the array-directed instantaneous frequency is related to the array-directed synthetic aperture angle $\theta_{A}$. Therefore, the array-directed angular resolution $\rho_{\theta }$ of the curved array SAR can be expressed as

(6) $$\begin{array}{ll} \rho_{\theta }= & \frac{2\pi }{\max\left\{f_{\theta }\right\}-\min\left\{f_{\theta }\right\}}\approx \frac{\lambda_{c}}{4R_{arc}(\theta_{A}/2)}\\ & \frac{\sqrt{R_{arc}^{2}+r_{n}^{2}-2R_{arc}r_{n}\cos\left(\theta -\theta_{n}\right)+{\left(H-h_{n}\right)}^{2}}}{r_{n}} \end{array}$$

where $f_{\theta }=\frac{4\pi f}{c}\frac{R_{arc}r_{n}\sin\left(\theta -\theta_{n}\right)}{\sqrt{R_{arc}^{2}+r_{n}^{2}-2R_{arc}r_{n}\cos\left(\theta -\theta_{n}\right)+{\left(H-h_{n}\right)}^{2}}}$.

## 3. Vibration Modelling Analysis

### 3.1. Signal Modelling under the Vibration Model

**Figure 3:**
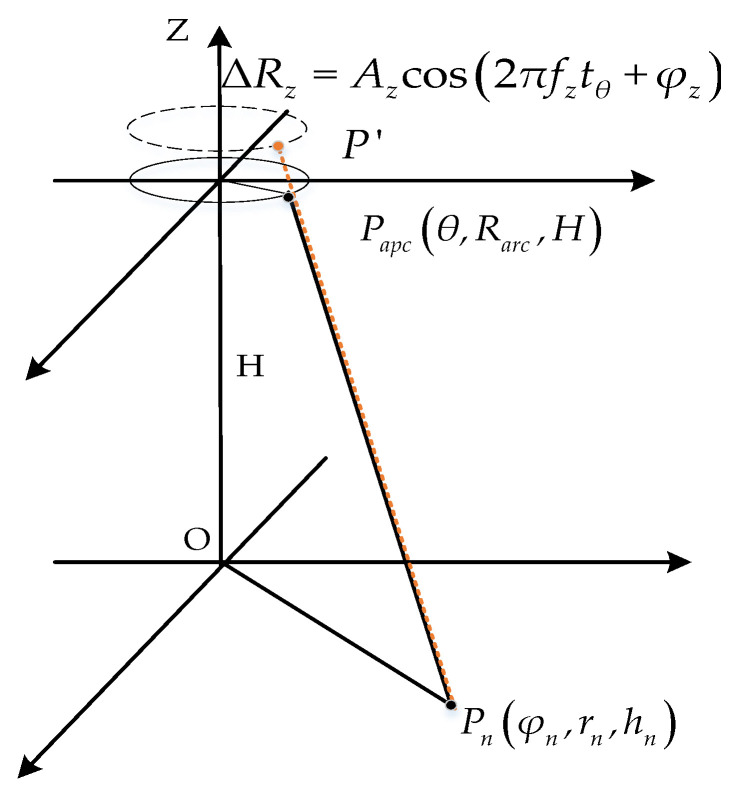
Simplified model of arc array vibration.

Let there be a unidirectional simple harmonic vibration of the airborne platform in the Z-axis direction $\Delta R_{z}=A_{z}\cos\left(2\pi f_{z}t_{\theta }+\varphi_{z}\right)$, then the simplified model of the vibration of the ideal scenario is shown in Figure 3. In Figure 3, the solid circle stands for the arc array antennas, the dashed circle stands for the arc array antennas after vibration and the red line stands for the instantaneous slant distance between the actual equivalent sampling point and the point target after vibration.

At this time, the instantaneous slant distance between the actual equivalent sampling point and the point target is

(7) $$R_{z}\left(\theta \right)=\sqrt{{\left(R_{arc}\cos\theta -r_{n}\cos\varphi_{n}\right)}^{2}+{\left(R_{arc}\sin\theta -r_{n}\sin\varphi_{n}\right)}^{2}+{\left(H-h+\Delta R_{z}\right)}^{2}}$$

Expanding and collating the above Taylor equation gives

(8) $$\begin{array}{ll} R_{z}\left(\theta \right) & =R_{arc}\sqrt{1+\frac{{r_{n}}^{2}+\Delta {R_{z}}^{2}-2R_{arc}r_{n}\cos\left(\theta -\varphi_{n}\right)+{\left(H-h\right)}^{2}+2\Delta R_{z}(H-h)}{{R_{arc}}^{2}}}\\ & \approx R_{arc}\left[1+\frac{{r_{n}}^{2}}{2{R_{arc}}^{2}}-\frac{2R_{arc}r_{n}\cos\left(\theta -\varphi_{n}\right)}{2{R_{arc}}^{2}}+\frac{{\left(H-h+\Delta R_{z}\right)}^{2}}{2{R_{arc}}^{2}})\right]\\ & =R_{arc}+\frac{{r_{n}}^{2}}{2R_{arc}}-r_{n}\cos\left(\theta -\varphi_{n}\right)+\frac{{\left(H-h+\Delta R_{z}\right)}^{2}}{2R_{arc}} \end{array}$$

The instantaneous slope distance formula under the error model is

(9) $$R_{z}^{\prime }\left(\theta \right)=R\left(\theta \right)+\frac{h-H}{R_{arc}}A_{z}\cos\left(2\pi f_{z}t_{\theta }+\varphi_{z}\right)$$

Substituting the expression for the slant distance into the echo signal gives

(10) $$\begin{array}{ll} ss_{re}\left(t,\theta \right)= & \sigma \left(\theta_{n},r_{n},h_{n}\right)\mathrm{rect}\left(\frac{t-R_{z}^{\prime }\left(\theta \right)/c}{T_{r}}\right)\mathrm{rect}\left(\frac{\theta -\varphi_{n}}{\theta_{a}}\right)\\ & \cdot \exp\left\{j2\pi \left[f_{c}\left(t-2R_{z}^{\prime }\left(\theta \right)/c\right)+\frac{1}{2}K_{r}{\left(t-\frac{2R_{z}^{\prime }\left(\theta \right)}{c}\right)}^{2}\right]\right\} \end{array}$$

The received signal is then mixed with the transmitted signal, and the intermediate frequency signal $ss_{IF}\left(t,\theta \right)$ is obtained by means of conjugate multiplication of the two signals

(11) $$\begin{array}{ll} ss_{IF}\left(t,\theta \right)= & \sigma \left(\theta_{n},r_{n},h_{n}\right)\mathrm{rect}\left(\frac{t-2R_{z}^{\prime }\left(\theta \right)}{T_{r}}\right)\mathrm{rect}\left(\frac{\theta -\varphi_{n}}{\theta_{a}}\right)\\ & \cdot \exp\left\{j2\pi \left[\left(f_{c}+K_{r}t\right)\frac{2R_{z}^{\prime }\left(\theta \right)}{c}-\frac{1}{2}K_{r}{\left(\frac{2R_{z}^{\prime }\left(\theta \right)}{c}\right)}^{2}\right]\right\} \end{array}$$

Compensation for the residual video phase (RVP) can obtain

(12) $$ss\left(t,\theta \right)=\sigma \left(\theta_{n},r_{n},h_{n}\right)\mathrm{rect}\left(\frac{t-2R_{z}^{\prime }\left(\theta \right)}{T_{r}}\right)\mathrm{rect}\left(\frac{\theta -\varphi_{n}}{\theta_{a}}\right)\cdot \exp\left\{j2\pi \left[\left(f_{c}+K_{r}t\right)\frac{2R_{z}^{\prime }\left(\theta \right)}{c}\right]\right\}$$

Collating the above equations gives

(13) $$\begin{array}{ll} ss\left(t,\theta \right)= & \sigma \left(\theta_{n},r_{n},h_{n}\right)\mathrm{rect}\left(\frac{t-2R_{z}^{\prime }\left(\theta \right)}{T_{r}}\right)\mathrm{rect}\left(\frac{\theta -\varphi_{n}}{\theta_{a}}\right)\cdot \exp\left[j4\pi R\left(\theta \right)\cdot \left(f_{c}+K_{r}t\right)\right]\\ & \cdot \exp\left[j4\pi \left(f_{c}+K_{r}t\right)\frac{(h-H)A_{z}\cos\left(2\pi f_{z}t_{\theta }+\varphi_{z}\right)}{cR_{arc}}\right] \end{array}$$

It follows that the second exponential term is the error term due to vibration, so let

(14) $$P\left(t,\theta \right)=\sigma \left(\theta_{n},r_{n},h_{n}\right)\mathrm{rect}\left(\frac{t-2R_{z}^{\prime }\left(\theta \right)}{T_{r}}\right)\mathrm{rect}\left(\frac{\theta -\varphi_{n}}{\theta_{a}}\right)\cdot \exp\left[j4\pi R\left(\theta \right)\cdot \left(f_{c}+K_{r}t\right)\right]$$

Let the phase error be

(15) $$M_{Z}=4\pi \left(f_{c}+K_{r}t\right)\frac{(h-H)A_{z}}{cR_{arc}}$$

So,

(16) $$P_{e}\left(t,\theta \right)=P\left(t,\theta \right)\cdot \exp\left[-jM_{Z}\cos\left(2\pi f_{z}t_{\theta }+\varphi_{z}\right)\right]$$

It follows from the first type of Bessel function that

(17) $$e^{jm_{f}\sin\Omega t}=\sum_{v=-\infty }^{+\infty }J_{v}\left(m_{f}\right)e^{jv\Omega t}$$

(18) $$J_{-v}\left(m_{f}\right)={\left(-1\right)}^{v}J_{v}\left(m_{f}\right)$$

Bringing in the echo signal gives

(19) $$\begin{array}{l} ss\left(t,\theta \right)=P\left(t,\theta \right)\left[\sum_{n=-\infty }^{+\infty }j^{n}J\left(M_{Z}\right)\exp\left(j2n\pi f_{z}t_{\theta }+\varphi_{z}\right)\right]\\ =P\left(t,\theta \right)\left\{J_{0}\left(M_{Z}\right)+\sum_{n=1}^{+\infty }J_{n}\left(M_{Z}\right)\left[\exp jn\left(2\pi f_{z}t_{\theta }+\varphi_{z}\right)+{\left(-1\right)}^{n}\exp-jn\left(2\pi f_{z}t_{\theta }+\varphi_{z}\right)\right]\right\} \end{array}$$

where $J_{n}\left(M_{Z}\right)$ is the *n*th order Bessel function coefficient.

The *n*th order error received signal $p_{z}\left(t;n\right)$ can be expressed by

(20) $$p_{z}\left(t;n\right)=P\left(t,\theta \right)\exp\left(j2\pi nf_{z}t_{\theta }\right)$$

So, the echo signal can be expressed by

(21) $$ss\left(t,\theta \right)=P\left(t,\theta \right)J_{0}\left(M_{Z}\right)+\sum_{n=1}^{+\infty }J_{n}\left(M_{Z}\right)\left[p_{z}\left(t:n\right)e^{jn\varphi_{z}}+{\left(-1\right)}^{n}p_{z}\left(t:-n\right)e^{-jn\varphi_{z}}\right]$$

Signals *p_z_*(*t*; *n*) and *p_z_*(*t*; *−n*) are the *n*th pair of paired echoes, and $J_{0}\left(M_{Z}\right)$ represents the main flap of the signal at the center of the azimuthal sampling, with an amplitude gain of $J_{n}\left(M_{Z}\right)$. Therefore, antenna vibration results in the attenuation of the main flap with infinite pairs of paired echoes.

### 3.2. Vibration Impact Analysis

#### 3.2.1. Direction of Vibration

Simple harmonic vibration in all directions ultimately manifests itself in the form of signals affecting the instantaneous slant distance between the antenna platform and the measured target point, and all are in the form of ideal slant distance plus the amount of change in slant distance. In the absence of vibration compensation, it can be assumed that every direction of vibration results in a large change in the instantaneous slant distance, and the impact of vibration in that direction on the imaging quality will be great. Figure 3 presents a simplified vibration model of the ideal scene, where there is a simple harmonic motion in the Z-axis $\Delta R_{z}=A_{z}\cos(2\pi f_{z}t_{\theta }+\varphi_{z})$; at this time, the actual equivalent sampling point *P*^′^ and the point target $P_{n}$ of the instantaneous slant distance is

(22) $$R_{z}\left(\theta \right)=\sqrt{{\left(R_{arc}\cos\theta -r_{n}\cos\varphi_{n}\right)}^{2}+{\left(R_{arc}\sin\theta -r_{n}\sin\varphi_{n}\right)}^{2}+{\left(H-h+\Delta R_{z}\right)}^{2}}$$

The Taylor expansion is shown in Equation (5), and compared to the ideal slant distance, the change in slant distance is

(23) $$\begin{array}{l} \Delta R_{z}\left(\theta \right)=R\left(\theta \right)-R_{z}\left(\theta \right)=R_{arc}+\frac{{r_{n}}^{2}}{2R_{arc}}-r_{n}\cos\left(\theta -\varphi_{n}\right)+\frac{{\left(H-h\right)}^{2}}{2R_{arc}}\\ -\left[R_{arc}+\frac{{r_{n}}^{2}}{2R_{arc}}-r_{n}\cos\left(\theta -\varphi_{n}\right)+\frac{{\left(H-h+\Delta R_{z}\right)}^{2}}{2R_{arc}}\right]\\ =\frac{{\left(H-h\right)}^{2}}{2R_{arc}}-\frac{{\left(H-h+\Delta R_{z}\right)}^{2}}{2R_{arc}}=\frac{2\Delta R_{z}\left(h-H\right)-\Delta {R_{z}}^{2}}{2R_{arc}}=\frac{\Delta R_{z}\left(h-H\right)}{R_{arc}} \end{array}$$

Similarly, it can be seen that the instantaneous change in the slope distance of the X-axis and the Y-axis is

(24) $$\Delta R_{x}\left(\theta \right)=\frac{\Delta R_{z}\left(R_{arc}\sin\theta +r_{n}\sin\varphi_{n}\right)}{R_{arc}}$$

(25) $$\Delta R_{y}\left(\theta \right)=\frac{\Delta R_{z}\left(R_{arc}\cos\theta +r_{n}\cos\varphi_{n}\right)}{R_{arc}}$$

Comparison leads to the observation that

(26) $$\Delta R_{z}\left(\theta \right)>\Delta R_{y}\left(\theta \right)>\Delta R_{x}\left(\theta \right)$$

From Equation (23), when simple harmonic vibration of the same magnitude occurs in all three directions, the change in slant distance in the direction of the Z-axis is the largest, so the vibration has the greatest effect on the Z-axis, followed by the Y-axis.

According to the simulation results, when the same size of simple harmonic vibration occurs in the three axes, it can be clearly seen that vibration in the direction of the Z-axis produces the largest number of pairs of echoes, and the imaging effect is the worst, followed by when there is vibration in the Y-axis, while when there is vibration in the direction of the X-axis, the imaging effect is relatively better, with almost no effect, and the theoretical derivation of the results is consistent. Therefore, this paper mainly focuses on the theoretical and simulation analysis of the existence of simple harmonic motion in the direction of the Z-axis.

#### 3.2.2. Amplitude of Vibration

From the above analysis, the echo signal under the vibration model can be expressed as

(27) $$\begin{array}{l} ss\left(t,\theta \right)=P\left(t,\theta \right)\left[\sum_{n=-\infty }^{+\infty }j^{n}J\left(M_{Z}\right)\exp\left(j2n\pi f_{z}t_{\theta }+\varphi_{z}\right)\right]\\ =P\left(t,\theta \right)\left\{J_{0}\left(M_{Z}\right)+\sum_{n=1}^{+\infty }J_{n}\left(M_{Z}\right)\left[\begin{array}{l} \exp\left(jn\left(2\pi f_{z}t_{\theta }+\varphi_{z}\right)\right)\\ +{\left(-1\right)}^{n}\exp\left(-jn\left(2\pi f_{z}t_{\theta }+\varphi_{z}\right)\right) \end{array}\right]\right\} \end{array}$$

where $\;J_{n}(\cdot )$ is the nth-order Bessel function and n denotes the number of main flaps of the signal and spurious paired echoes. $\;J_{n}(\cdot )$ trends toward zero as *n* increases. The vibrational phase introduced by the platform vibration leads to multiple Doppler shifts, which replicate the original signal and produce multiple pairs of paired echoes. The echo signal array-wise envelope is modulated by a Type I Bessel function.

**Figure 4:**
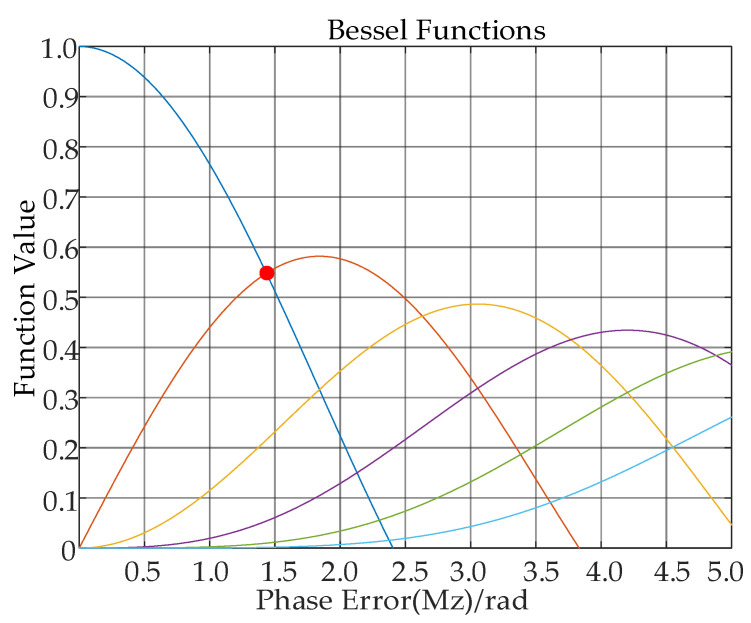
0th to 6th order Bessel functions.

From Figure 4, the different lines stand for the 0th to 6th order Bessel functions. The red dot stands for $M_{z}=1.4$, it can be seen that as $\;M_{z}$ varies, the overall oscillation of $\;J_{n}\left(M_{z}\right)$ trends toward 0, and when $M_{z}<1.4$, as the order increases, $J_{n}\left(M_{z}\right)$ decreases gradually. Therefore, the amplitude of vibration not only affects the peak value of the main image, but also affects the number of echoes. The larger the amplitude, the more pairs of echoes are generated.

#### 3.2.3. Frequency of Vibration

Using the Doppler parametric model can obtain

(28) $$R\left(t\right)=r_{0}+r_{1}t+r_{2}t^{2}+r_{3}t^{3}+r_{4}t^{4}$$

(29) $$R\left(t\right)=R_{z}\left(\theta \right)$$

So,

(30) $$r_{0}+r_{1}t+r_{2}t^{2}+r_{3}t^{3}+r_{4}t^{4}=R\left(\theta \right)+\frac{h-H}{R_{arc}}A_{z}\cos\left(2\pi f_{z}t_{\theta }+\varphi_{z}\right)$$

A Taylor’s formula expansion of the right-hand side equation yields

(31) $$\begin{array}{ll} R_{z}\left(\theta \right) & =R\left(\theta \right)+\frac{h-H}{R_{arc}}A_{z}\cos\left(2\pi f_{z}t_{\theta }+\varphi_{z}\right)\\ & =R\left(\theta \right)+\frac{h-H}{R_{arc}}A_{z}\left[\cos2\pi f_{z}t_{\theta }\cos\varphi_{z}-\sin2\pi f_{z}t_{\theta }\sin\varphi_{z}\right]\\ & =R\left(\theta \right)+\frac{h-H}{R_{arc}}A_{z}\cos\varphi_{z}\left[1-\frac{1}{2}{\left(2\pi f_{z}t_{\theta }\right)}^{2}+\frac{1}{4!}{\left(2\pi f_{z}t_{\theta }\right)}^{4}+o\left(t_{\theta }^{4}\right)\right]\\ & -\frac{h-H}{R_{arc}}A_{z}\sin\varphi_{z}\left[2\pi f_{z}t_{\theta }-\frac{1}{3!}{\left(2\pi f_{z}t_{\theta }\right)}^{3}+o\left(t_{\theta }^{3}\right)\right] \end{array}$$

Solving the equation gives

(32) $$\left\{ \begin{array}{l} r_{0}=R\left(\theta \right)+\frac{h-H}{R_{arc}}A_{z}\cos\varphi_{z}\\ r_{1}=-2\pi f_{z}\frac{h-H}{R_{arc}}A_{z}\sin\varphi_{z}\\ r_{2}=-2\pi^{2}{f_{z}}^{2}\frac{h-H}{R_{arc}}A_{z}\cos\varphi_{z}\\ r_{3}=\frac{4}{3}\pi^{3}{f_{z}}^{3}\frac{h-H}{R_{arc}}A_{z}\sin\varphi_{z}\\ r_{4}=\frac{2}{3}\pi^{4}{f_{z}}^{4}\frac{h-H}{R_{arc}}A_{z}\cos\varphi_{z} \end{array}\right.$$

The nth order error of the received signal is

(33) $$p_{z}\left(t;n\right)=P\left(t,\theta \right)\exp\left(j2\pi nf_{z}t_{\theta }\right)$$

The spectrum of the error received signal can be expressed as

(34) $$P_{z}\left(f_{d}\right)=P\left(f_{d}+nf_{z}\right)$$

where $f_{a}$ is the Doppler frequency and $f_{d}$ is the Doppler frequency when the Doppler center frequency $f_{dc}$ is removed.

(35) $$f_{d}=f_{a}-f_{dc}$$

Therefore, the error of the received signal for the frequency domain matching response can be expressed as

(36) $$\begin{array}{l} s\left(t;n\right)=\int_{-\infty }^{+\infty }P\left(f_{d}+nf_{z}\right)H\left(f_{d}\right)\exp\left(j2\pi f_{a}\right)df_{a}\\ =\int_{-\infty }^{+\infty }g\left(t_{f}\right)\exp\left[-j2\pi \left(E_{0}+E_{1}f_{d}+E_{2}{f_{d}}^{2}+E_{3}{f_{d}}^{3}\right)\right]\exp\left(j2\pi f_{a}t\right)df_{a} \end{array}$$

where $t_{f}$ is the Doppler frequency $\;f_{d}$ corresponding to the azimuth time and $\;g\left(t_{f}\right)$ is the antenna gain. $\;E_{0}\sim E_{3}$ is the zero-order to third-order error coefficient of the matching phase error, which can be expressed as

(37) $$\left\{ \begin{array}{l} E_{0}=\frac{1}{4}F_{1}\lambda {\left(nf_{z}\right)}^{2}+\frac{1}{12}F_{2}\lambda^{2}{\left(nf_{z}\right)}^{3}+\frac{1}{32}F_{3}\lambda^{3}{\left(nf_{z}\right)}^{4}\\ E_{1}=-\frac{1}{2}F_{1}\lambda \left(nf_{z}\right)-\frac{1}{4}F_{2}\lambda^{2}{\left(nf_{z}\right)}^{2}-\frac{1}{8}F_{3}\lambda^{3}{\left(nf_{z}\right)}^{3}\\ E_{2}=\frac{1}{4}F_{2}\lambda^{2}\left(nf_{z}\right)+\frac{3}{16}F_{3}\lambda^{3}{\left(nf_{z}\right)}^{2}\\ E_{3}=-\frac{1}{8}F_{3}\lambda^{3}\left(nf_{z}\right) \end{array}\right.$$

The spectral factor $F_{1}\sim F_{3}$ can be expressed as

(38) $$\left\{ \begin{array}{l} F_{1}=\frac{1}{2r_{2}}\\ F_{2}=-\frac{3r_{3}}{8r_{2}^{3}}\\ F_{3}=\frac{9r_{3}^{2}-4r_{2}r_{4}}{16{r_{2}}^{5}} \end{array}\right.$$

Therefore, the point spread function under the influence of antenna displacement vibration can be expressed as

(39) $$s_{e}\left(t\right)=J_{0}\left(M_{z}\right)s\left(t\right)+\sum_{n=1}^{\infty }J_{n}\left(M_{z}\right)\left[s_{v}\left(t;n\right)e^{-jn\varphi_{z}}+{\left(-1\right)}^{n}s_{v}\left(t;-n\right)e^{jn\varphi_{z}}\right]$$

where *s*(*t*) is the ideal point expansion function.

According to the frequency domain matched response function, it can be seen that the antenna vibration phase induces the Doppler phase error of the paired echoes. In conventional airborne SAR, the azimuth spectrum is of low order, which only generates zero-order and first-order Doppler phase errors. The zero-order phase error leads to a constant phase error of the point-pair echoes, and the first-order phase error leads to an azimuthal shift in the pair echoes with respect to the ideal point expansion function, and does not lead to the scattering of the pair echoes.

The azimuthal time offset of the *n*th pair of paired echoes with respect to the position of the main flap of the point spread function is expressed as

(40) $$E_{1}=-t_{f}\left(\pm nf_{z}\right)\approx \pm \frac{nf_{z}}{f_{dr}}$$

where $\;f_{dr}$ is the Doppler modulation frequency and the amplitude of the paired echoes with respect to the main flap of the point spread function is

(41) $$A_{z}=\frac{C_{r}\left(nf_{z}\right)J_{n}\left(M_{z}\right)}{J_{0}\left(M_{z}\right)}$$

where $C_{r}\left(nf_{z}\right)$ is the amplitude attenuation of the paired echoes due to second- and third-order phase errors, which is positively correlated with the vibration frequency $\;nf_{z}$. The paired echoes can be expressed as

(42) $$s\left(t;n\right)=\exp(-j2\pi E_{0})s\left(t-E_{1}\right)$$

If the antenna is uniformly weighted, the time after offset is

(43) $$t_{e}=t-E_{1}$$

The paired echoes can be expressed as:

(44) $$s_{v}\left(t;n\right)\approx \exp\left[j2\pi \left(f_{dc}t-E_{0}\right)\right]B_{a}\cdot \left\{\begin{array}{l} \{\sin c\left(B_{a}t_{e}\right)-j\frac{E_{2}}{\pi t_{e}}\left[1-\sin c\left(B_{a}t_{e}\right)\right]\cos\left(\pi B_{a}t_{e}\right)\\ +\frac{E_{3}}{\pi^{2}t_{e}^{3}}\left[2+\cos\left(\pi B_{a}t_{e}\right)-3\sin c\left(B_{a}t_{e}\right)\right]\cos\left(\pi B_{a}t_{e}\right) \end{array}\right\}$$

Therefore, the higher the vibration frequency, the greater the second- and third-order Doppler phase errors, and consequently the greater the amplitude attenuation of the paired echoes, and the further away the paired echoes will be from the main image.

## 4. Vibration Compensation Algorithm

According to the above simulation results, the on-board antenna platform will cause a sinusoidal phase error term in the return signal of the arc array after distance compression. The simple harmonic vibration of the antenna platform will change the echo signal from linear frequency modulated (LFM) to sinusoidal frequency modulation (SFM) [23]. Therefore, the imaging algorithm for ideal scenarios is no longer suitable for vibrating arc array SAR imaging. In this paper, we propose a vibration phase estimation and compensation algorithm based on delay conjugate multiplication. Firstly, the distance pulse pressure processing is performed on the echo signal; secondly, conjugate multiplication of the pulse pressure signal and its delay within the same distance unit is performed, and the phase of the signal after conjugate multiplication is extracted, and then the amplitude and phase compensation on the extracted phase is performed, so as to estimate the vibration phase. Finally, the vibration phase is compensated for in the azimuthal direction for the distance pulse pressure signal to eliminate the paired echoes [24]. This algorithm does not require any a priori knowledge and does not introduce new phase errors at the same time, and it does not require isolated scattering points.

### Algorithmic Process

The echo signal is known to be

(45) $$\begin{array}{ll} ss\left(t,\theta \right)= & \sigma \left(\theta_{n},r_{n},h_{n}\right)\mathrm{rect}\left(\frac{t-2R_{z}^{\prime }\left(\theta \right)}{T_{r}}\right)\mathrm{rect}\left(\frac{\theta -\varphi_{n}}{\theta_{a}}\right)\cdot \exp\left[\frac{j4\pi R\left(\theta \right)\cdot \left(f_{c}+K_{r}t\right)}{c}\right]\\ & \cdot \exp\left[j4\pi \left(f_{c}+K_{r}t\right)\frac{(h-H)A_{z}\cos\left(2\pi f_{z}t_{\theta }+\varphi_{z}\right)}{cR_{arc}}\right] \end{array}$$

According to the Bessel function

(46) $$\begin{array}{l} ss\left(t,\theta \right)=P\left(t,\theta \right)\left[\sum_{n=-\infty }^{+\infty }j^{n}J\left(M_{Z}\right)\exp\left(j2n\pi f_{z}t_{\theta }+\varphi_{z}\right)\right]\\ =P\left(t,\theta \right)\left\{J_{0}\left(M_{Z}\right)+\sum_{n=1}^{+\infty }J_{n}\left(M_{Z}\right)\left[\exp jn\left(2\pi f_{z}t_{\theta }+\varphi_{z}\right)+{\left(-1\right)}^{n}\exp-jn\left(2\pi f_{z}t_{\theta }+\varphi_{z}\right)\right]\right\} \end{array}$$

Since $t_{\theta }$ in the arc array SAR can be equated with *θ*,

(47) $$t_{\theta }=\theta \Delta T_{\theta }/\Delta \theta$$

The Fourier transform of the above equation in the azimuthal direction yields

(48) $$\begin{array}{l} S\left(f_{r},f_{\theta }\right)=\sigma_{n}\left(\theta_{n},r_{n},h_{n}\right)w_{r}\left(\frac{f-f_{c}}{B}\right)w_{\theta }\left(g\right)\cdot \left\{\sum_{-\infty }^{+\infty }J_{n}\left(a\right)\exp\left(jn\varphi_{n}\right)\right\}\\ \cdot \exp\left\{\begin{array}{l} \frac{j4\pi \left(f_{c}+f_{r}\right)R\left(0\right)}{c}+\frac{j\pi R\left(0\right)c\Delta T_{\theta }}{2R_{arc}r_{n}\left(f_{c}+f_{r}\right)\Delta \theta }f_{\theta }^{2}\\ -\frac{jn\pi R\left(0\right)cf_{z}\Delta T_{\theta }}{2R_{arc}r_{n}\left(f_{c}+f_{r}\right)\Delta \theta }f_{\theta }+\frac{jn^{2}\pi R\left(0\right)cf_{z}\Delta T_{\theta }}{2R_{arc}r_{n}\left(f_{c}+f_{r}\right)\Delta \theta }f_{z}^{2} \end{array}\right\} \end{array}$$

This is obtained by keeping the first three terms and combining all constant terms:

(49) $$\begin{array}{l} S\left(f_{r},f_{\theta }\right)=\sigma_{n}\left(\theta_{n},r_{n},h_{n}\right)w_{r}\left(\frac{f-f_{c}}{B}\right)w_{\theta }\left(g\right)\cdot \left\{\sum_{-\infty }^{+\infty }J_{n}\left(a\right)\exp\left(jn\varphi_{n}\right)\right\}\\ \cdot \exp\left\{\frac{j4\pi f_{c}R\left(0\right)}{c}+\frac{jCR\left(0\right)}{2f_{c}}f_{\theta }^{2}-\frac{jnCR\left(0\right)cf_{z}}{f_{c}}f_{\theta }\right\} \end{array}$$

When the platform vibrates, the phase of the echo signal is $\varphi =A_{z}cos(2\pi f_{z}t_{\theta }+\varphi_{z})$, which is the modulation phase artificially introduced by the platform vibration, and causes the echo signal to generate pairs of echoes in the azimuthal direction, which needs to be compensated for. When |*φ*| > *π*, the phase itself will generate entanglement, and the phase extraction will be inaccurate. Therefore, the phase cannot be extracted directly, and the phase should be extracted by means of conjugate multiplication of the echo signal and its delay signal. The specific steps of vibration phase estimation and compensation are described below.

During delayed conjugate multiplication of signals from the same distance unit, we define the distance pulse pressure signal $s_{v}$ within a distance cell as the signal of interest (SOI):

(50) $$s_{v}=w\left(t_{\theta }\right)\cdot \exp\left(-j\frac{4\pi }{\lambda }\left(A_{z}\cos\left(2\pi f_{z}t_{\theta }+\varphi_{z}\right)\right)\right)$$

where $w\left(t_{\theta }\right)$ is the window function, and for $s_{v}$, the time delay *τ* can be obtained:

(51) $$s_{vd}=w\left(t_{\theta }-\tau \right)\cdot \exp\left(-j\frac{4\pi }{\lambda }\left(A_{z}\cos\left(2\pi f_{z}\left(t_{\theta }-\tau \right)+\varphi_{z}\right)\right)\right)$$

We make the two conjugations and multiply them:

(52) $$s_{v}s_{vd}^{*}=w^{2}\left(t_{m}\right)\exp\left(-j\frac{4\pi }{\lambda }A_{z}\cdot 2\cos\left(\frac{f_{z}\tau }{2}\right)\sin\left(f_{z}\left(t_{\theta }-\frac{\tau }{2}\right)+\varphi_{z}\right)\right)$$

We then extract the vibration phase:

(53) $$\begin{array}{ll} \varphi_{v} & =\left(-j\frac{4\pi }{\lambda }A_{z}\cdot 2cos\left(\frac{f_{z}\tau }{2}\right)\sin\left(f_{z}\left(t_{\theta }-\frac{\tau }{2}\right)+\varphi_{z}\right)\right)\\ & =\left(-j\frac{4\pi }{\lambda }A_{z}\cdot 2cos\left(\frac{f_{z}\tau }{2}\right)\cos\left(f_{z}\left(t_{\theta }-\frac{\tau }{2}\right)+\varphi_{z}-\frac{\pi }{2}\right)\right)\\ & =\left(-j\frac{4\pi }{\lambda }A_{z}\cdot 2cos\left(\frac{f_{z}\tau }{2}\right)\cos\left(f_{z}\left(t_{\theta }-\frac{\tau }{2}-\frac{\pi }{2f_{z}}\right)+\varphi_{z}\right)\right) \end{array}$$

Compared to the original phase, the vibrational phase has an additional amplitude modulation term of $2\cos\left(f_{z}\tau /2\right)$ and a time delay term of $f_{z}\left(-\tau /2-\pi /2f_{z}\right)$, which can be compensated for to recover the original phase.

Eliminating the amplitude modulation and displacement terms, a Fourier transform of the vibrational phase is obtained:

(54) $$F\left(\varphi_{v}\right)=-\frac{4\pi }{\lambda }A_{z}\cdot 2\cos\left(\frac{f_{z}\tau }{2}\right)\left(\delta \left(f_{\theta }+f_{v}\right)+\delta \left(f_{\theta }-f_{v}\right)\right)\cdot \exp\left(-j2\pi f_{\theta }\left(\frac{\tau }{2}-\frac{\pi }{2f_{z}}\right)\right)$$

The last exponential term is the displacement term. We multiply the above equation by $\exp\left(j2\pi f_{\theta }\left(\tau /2-\pi /2f_{z}\right)\right)/2\cos\left(f_{z}\tau /2\right)$ to remove the amplitude modulation term and the displacement term:

(55) $$F\left(\varphi_{v}\right)=-\frac{4\pi }{\lambda }A_{z}\left(\delta \left(f_{\theta }+f_{v}\right)+\delta \left(f_{\theta }-f_{v}\right)\right)$$

To estimate the phase of vibration, we perform an inverse Fourier transform of the above equation to produce an estimate of the vibration phase ${\varphi^{\prime }}_{v}$,

(56) $$\varphi_{v}^{\prime }=-\frac{4\pi }{\lambda }A_{z}\cos\left(f_{z}t_{\theta }+\varphi_{v}^{\prime \prime }\right)$$

We compensate for the vibrational phase by compensating for the distance pulse pressure signal using the estimated phase error ${\varphi^{\prime }}_{v}$ until the vibrational phase is negligible for imaging. Multiplying $s_{v}$ by $\;\exp\left(-j{\varphi^{\prime }}_{v}\right)$ gives the first compensation result:

(57) $$\begin{array}{l} s_{1}=s_{v}*\exp\left(-j\varphi_{v}^{\prime }\right)\\ =w\left(t_{\theta }\right)\cdot \exp\left(j\varphi_{c}-j\frac{4\pi }{\lambda }\right)A_{z}\cos\left(f_{z}t_{\theta }+\varphi_{z}\right)+j\frac{4\pi }{\lambda }A_{z}\cos\left(f_{z}t_{\theta }+\varphi_{v}^{\prime \prime }\right)\\ =w\left(t_{\theta }\right)\cdot \exp\left(j\varphi_{c}\right)\exp\left(-j\frac{4\pi }{\lambda }A_{z}\left[\cos\left(f_{z}t_{\theta }+\varphi_{z}\right)-\cos\left(f_{z}t_{\theta }+\varphi_{z}-\Delta \varphi_{z}\right)\right]\right)\\ =w\left(t_{\theta }\right)\cdot \exp\left(j\varphi_{c}\right)\exp\left(-j\frac{8\pi }{\lambda }A_{z}\cos\left(\frac{\Delta \varphi_{z}}{2}\right)\sin\left(f_{z}t_{\theta }+\varphi_{z}-\frac{\Delta \varphi_{z}}{2}\right)\right)\\ =w\left(t_{\theta }\right)\cdot \exp\left(j\varphi_{c}\right)\exp\left(-j\frac{8\pi }{\lambda }A_{z}\cos\left(\frac{\Delta \varphi_{z}}{2}\right)\sin\left(f_{z}t_{\theta }+\varphi_{z1}\right)\right) \end{array}$$

The residual error phase is

(58) $$\varphi_{c1}=-j\frac{8\pi }{\lambda }A_{z}\cos\left(\frac{\Delta \varphi_{z}}{2}\right)sin\left(f_{z}t_{\theta }+\varphi_{z1}\right)$$

We define the first pair of paired echoes with respect to the real target amplitude attenuation function as

(59) $$f\left(x\right)=20lg\frac{J_{1}\left(x\right)}{J_{0}\left(x\right)}$$

where $J_{0}\left(x\right)$ denotes the true amplitude, $J_{1}\left(x\right)$ denotes the amplitude of the first pair of paired echoes, *x* denotes $-4\pi A_{z}^{\prime }/\lambda$, and $A_{z}^{\prime }$ denotes the true phase amplitude before the vibration compensation $A_{z}^{\prime }=A_{z}$. When f(x)≤−30 dB, the paired echoes can be ignored.

**Figure 5:**
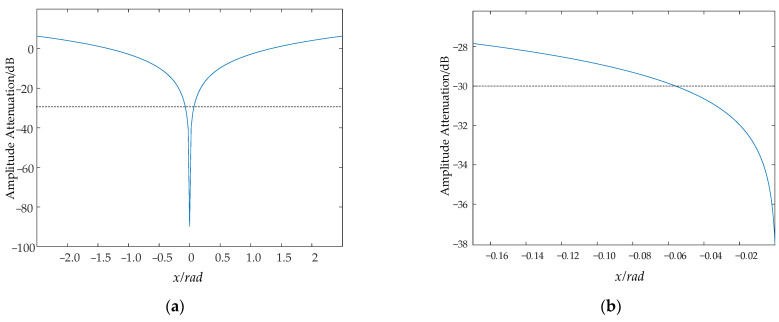
Attenuation function. (**a**) Attenuation function. (**b**) Partial enlargement.

We can see from Figure 5 that the blue line stands for attenuation function, when x≤0.06 rad, f(x)≤−30 dB.

**Figure 6:**
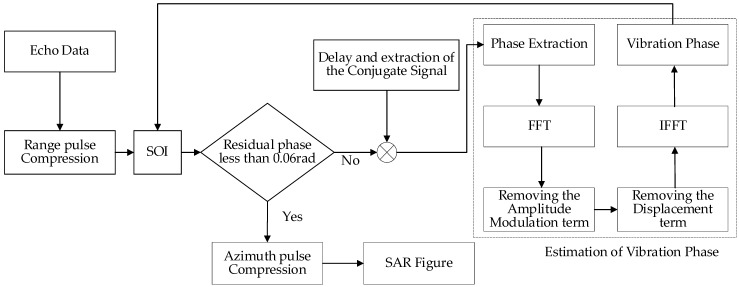
Algorithm flow diagram.

The compensation stops when it is less than 0.06 rad; otherwise, the iteration has to continue. The flowchart of the algorithm is shown below in Figure 6.

## 5. Simulation Analysis

In the imaging of arc array SAR, the vibration phase caused by the platform vibration will be infinitely copied in the azimuth direction, forming a pair of echoes, which seriously affects the imaging quality of SAR images. The larger the amplitude, the greater the number of paired echoes generated; the higher the vibration frequency is, the further the paired echo is from the main image. Based on the above theoretical derivation, this section simulates the above theory. The simulation parameters are shown in Table 1. Firstly, the influence of the error is simulated and analyzed to verify the influence of vibration direction, vibration amplitude and vibration frequency on vibration. In addition, the correction algorithm is verified, which proves the effectiveness of the compensation algorithm.

### 5.1. Vibration Impact Analysis

**Figure 7:**
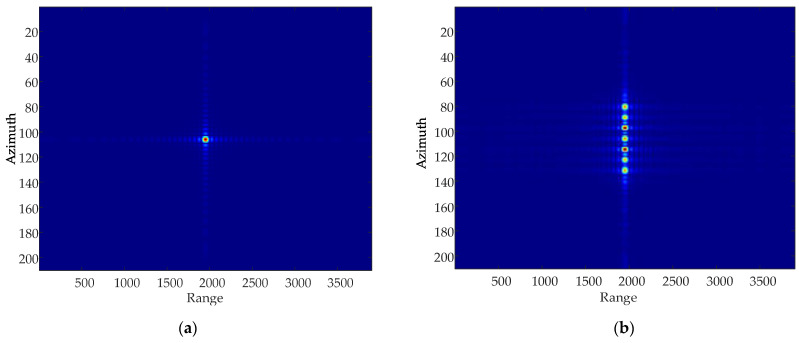
Point target azimuthal simulation. (**a**) Point target imaging results without the vibrational phase. (**b**) Point target imaging results when adding the vibrational phase.

Due to the influence of paired echoes, the point target is copied many times in the azimuth direction, resulting in the real target not being able to be identified. As shown in Figure 7 the colorful points stand for the point target. In Figure 7a, imaging result without adding the vibrational phase only shows the real target, while Figure 7b presents the pair of false targets in addition to the real target after adding the vibrational phase. This result shows that the sinusoidal modulation phase introduced by the vibrational phase error produces false targets in the azimuthal direction, also known as “ghost images”.

#### 5.1.1. Direction of Vibration

Based on the simulation parameters in Table 1, this paper simulates and analyses the case where there are simple harmonic motions with the same amplitude, frequency and phase in the X-axis, Y-axis and Z-axis, respectively, and obtains contour maps and azimuthal slices of a single point target.

**Figure 8:**
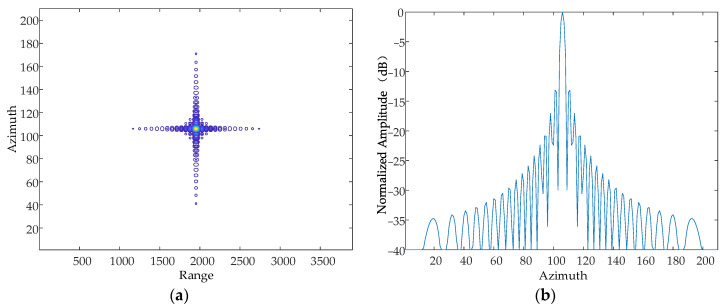
Contour map and azimuthal slices of the X-axis. (**a**) Contour plot, whose horizontal coordinates represent the number of distance-directed sampling points and whose vertical coordinates represent the number of array-directed sampling points, and the contour plot reflects the focusing performance of the point target. (**b**) Array-direction response function, with the horizontal coordinates representing the number of array-direction sampling points and the vertical coordinates representing the normalized amplitude, and the array-direction slices reflect the number of pairs of echoes generated by the vibration.

**Figure 9:**
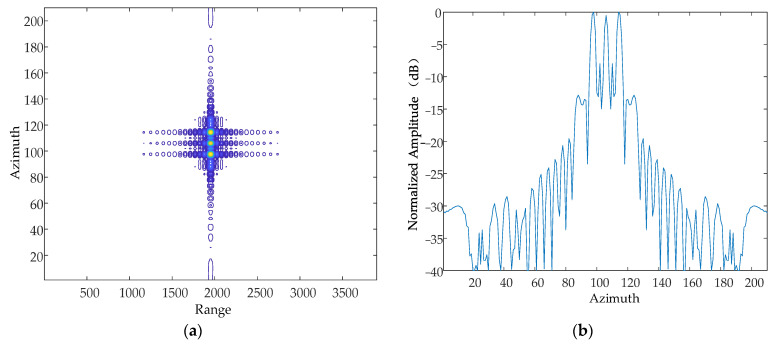
Contour map and azimuthal slices of the Y-axis. (**a**) Contour plot, whose horizontal coordinates represent the number of distance-directed sampling points and whose vertical coordinates represent the number of array-directed sampling points, and the contour plot reflects the focusing performance of the point target. (**b**) Array-direction response function, with the horizontal coordinates representing the number of array-direction sampling points and the vertical coordinates representing the normalized amplitude, and the array-direction slices reflect the number of pairs of echoes generated by the vibration.

**Figure 10:**
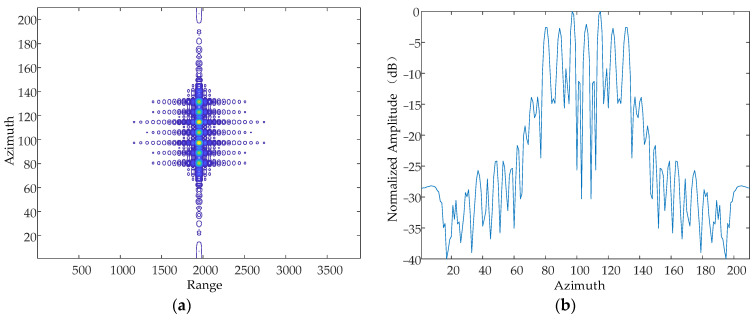
Contour map and azimuthal slices of *Z*-axis. (**a**) Contour plot, whose horizontal coordinates represent the number of distance-directed sampling points and whose vertical coordinates represent the number of array-directed sampling points, and the contour plot reflects the focusing performance of the point target. (**b**) Array-direction response function, with the horizontal coordinates representing the number of array-direction sampling points and the vertical coordinates representing the normalized amplitude, and the array-direction slices reflect the number of pairs of echoes generated by the vibration.

According to the national military standards GJB 779-1989 “General specification for airborne electronic equipment chassis and mounting bracket” [22] and GJB150.16-86 “Vibration test of military equipment environmentally applicable method”, the vibration frequency range caused by a helicopter itself is generally in the range of 10~500 Hz, and the range of the vibration amplitude is approximately in the range of 0~10 mm. Based on this, this paper sets the vibration frequency as 500 Hz and the amplitude as 5 mm, and simulates the X-axis, Y-axis and Z-axis, respectively. The simulation results are as follows in Figure 8, Figure 9 and Figure 10.

#### 5.1.2. Amplitude of Vibration

**Figure 11:**
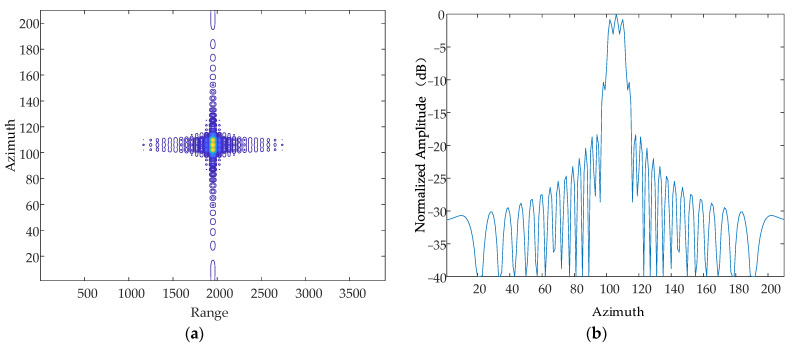
Contour map and azimuthal slices of 1 mm. (**a**) Contour plot, whose horizontal coordinates represent the number of distance-directed sampling points and whose vertical coordinates represent the number of array-directed sampling points, and the contour plot reflects the focusing performance of the point target. (**b**) Array-direction response function, with the horizontal coordinates representing the number of array-direction sampling points and the vertical coordinates representing the normalized amplitude, and the array-direction slices reflect the number of pairs of echoes generated by the vibration.

**Figure 12:**
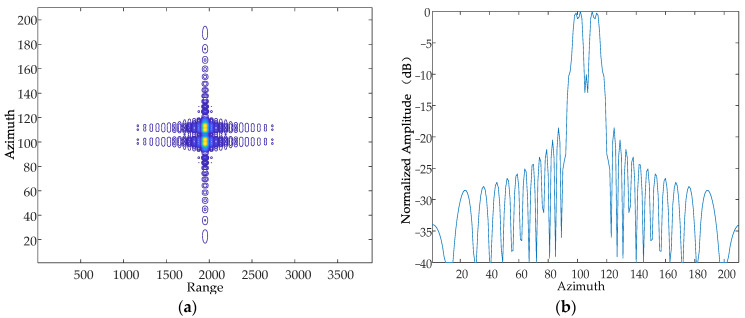
Contour map and azimuthal slices of 2 mm. (**a**) Contour plot, whose horizontal coordinates represent the number of distance-directed sampling points and whose vertical coordinates represent the number of array-directed sampling points, and the contour plot reflects the focusing performance of the point target. (**b**) Array-direction response function, with the horizontal coordinates representing the number of array-direction sampling points and the vertical coordinates representing the normalized amplitude, and the array-direction slices reflect the number of pairs of echoes generated by the vibration.

**Figure 13:**
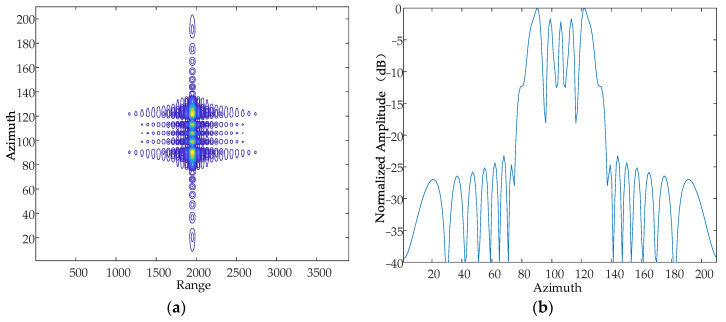
Contour map and azimuthal slices of 5 mm. (**a**) Contour plot, whose horizontal coordinates represent the number of distance-directed sampling points and whose vertical coordinates represent the number of array-directed sampling points, and the contour plot reflects the focusing performance of the point target. (**b**) Array-direction response function, with the horizontal coordinates representing the number of array-direction sampling points and the vertical coordinates representing the normalized amplitude, and the array-direction slices reflect the number of pairs of echoes generated by the vibration.

The amplitude of the simple harmonic motion in the direction of the Z-axis at a certain moment is 1 mm, 2 mm and 5 mm, respectively, and the vibration frequency is 10 Hz. The simulation results are as follows in Figure 11, Figure 12 and Figure 13.

From the simulation results, it can be seen that when the vibration frequency and vibration direction are kept constant, the vibration amplitude is increased sequentially, and the number of generated pairs of echoes increases gradually, which is consistent with the theoretical results.

#### 5.1.3. Frequency of Vibration

**Figure 14:**
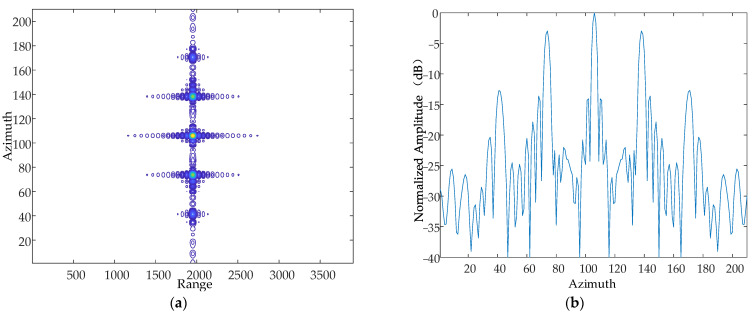
Contour map and azimuthal slices of 10 Hz. (**a**) Contour plot, whose horizontal coordinates represent the number of distance-directed sampling points and whose vertical coordinates represent the number of array-directed sampling points, and the contour plot reflects the focusing performance of the point target. (**b**) Array-direction response function, with the horizontal coordinates representing the number of array-direction sampling points and the vertical coordinates representing the normalized amplitude, and the array-direction slices reflect the number of pairs of echoes generated by the vibration.

**Figure 15:**
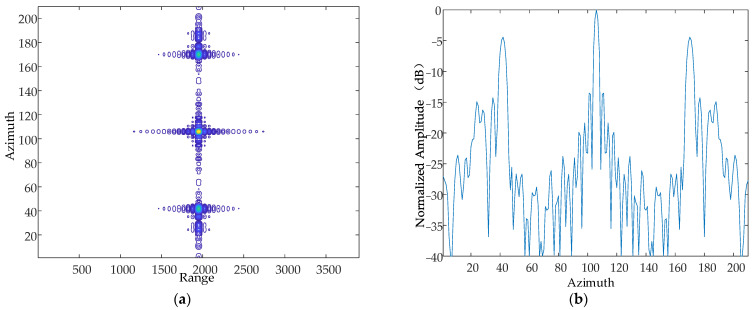
Contour map and azimuthal slices of 20 Hz. (**a**) Contour plot, whose horizontal coordinates represent the number of distance-directed sampling points and whose vertical coordinates represent the number of array-directed sampling points, and the contour plot reflects the focusing performance of the point target. (**b**) Array-direction response function, with the horizontal coordinates representing the number of array-direction sampling points and the vertical coordinates representing the normalized amplitude, and the array-direction slices reflect the number of pairs of echoes generated by the vibration.

**Figure 16:**
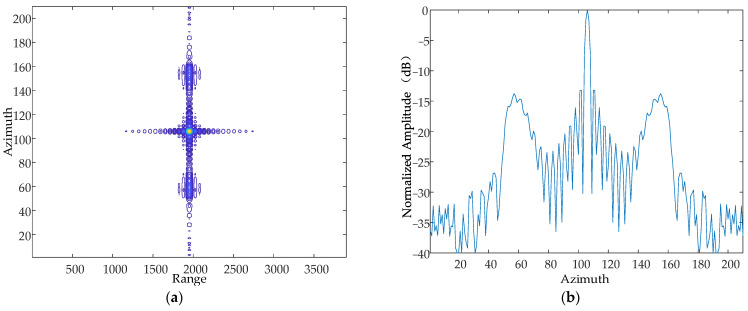
Contour map and azimuthal slices of 50 Hz. (**a**) Contour plot, whose horizontal coordinates represent the number of distance-directed sampling points and whose vertical coordinates represent the number of array-directed sampling points, and the contour plot reflects the focusing performance of the point target. (**b**) Array-direction response function, with the horizontal coordinates representing the number of array-direction sampling points and the vertical coordinates representing the normalized amplitude, and the array-direction slices reflect the number of pairs of echoes generated by the vibration.

Based on the simulation parameters in Table 1, this paper simulates and analyses the case where there are simple harmonic motions with the same amplitude and phase in the Z-axis, and the frequency of the simple harmonic motion in the direction of the Z-axis at a certain moment is 10 Hz, 50 Hz and 70 Hz, respectively. The simulation results are shown in Figure 14, Figure 15 and Figure 16.

From the simulation results, it can be seen that when the vibration amplitude and vibration direction are kept constant, the vibration frequency is increased sequentially, and the resulting pairs of echoes are further and further away from the main image, which is consistent with the theoretical results.

### 5.2. Algorithm Simulation

**Figure 17:**
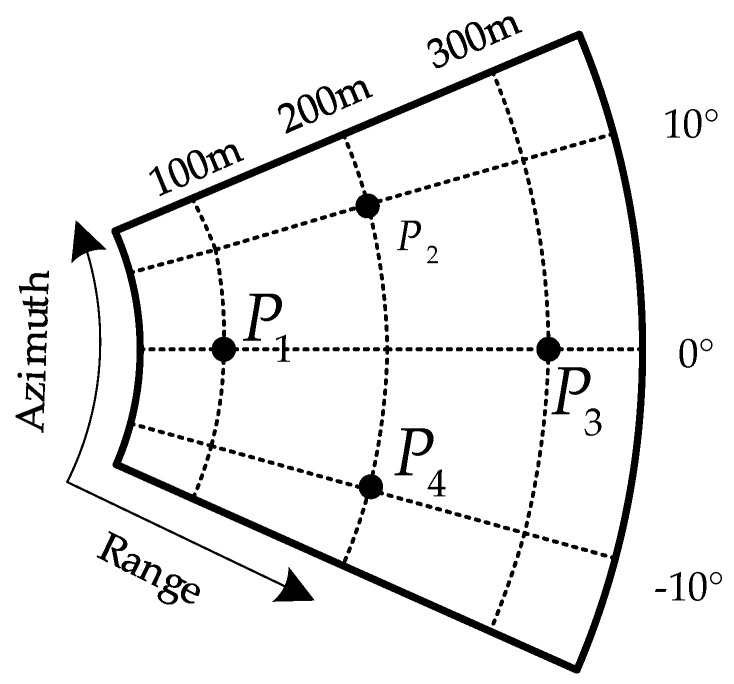
Point target distribution map.

In this section, four point targets are selected for simulation, and the real position of the point targets is shown in Figure 17. The dots in the figure stand for the actual position in the scene.

**Figure 18:**
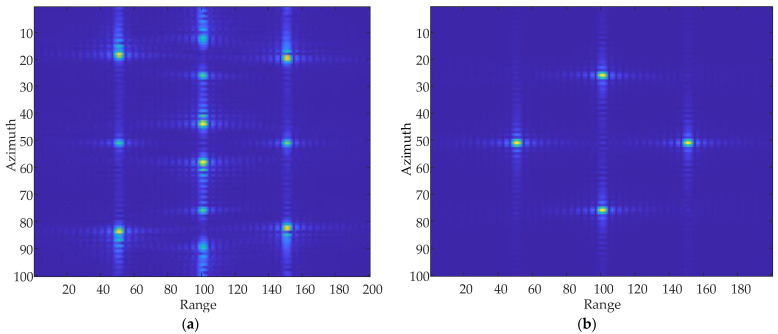
Focused images of multiple point targets. (**a**) Focused image before compensation. (**b**) Focused image after compensation.

The focused image of the point targets is obtained after processing using the algorithm, as shown in Figure 18. The dots in the figure stand for the actual position in the scene.

**Figure 19:**
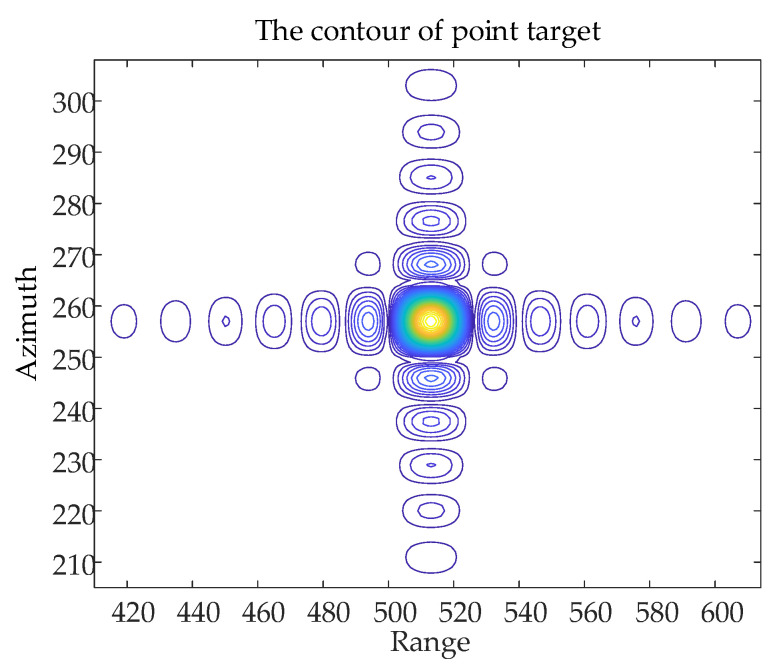
The contours of the point target *P*_1_.

**Figure 20:**
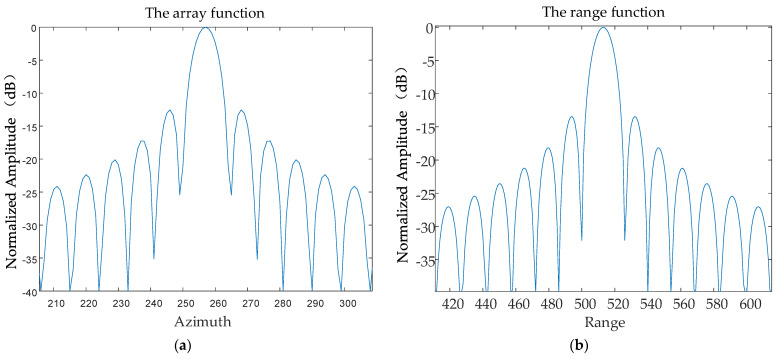
The response function of the point target *P*_1_. (**a**) The array function. (**b**) The range function.

The contours of the point target *P*_1_ are shown in Figure 19 and the array–direction and distance–direction response functions corresponding to the point target $P_{1}$ are shown in Figure 20.

Besides, the imaging performance analysis is shown in Table 2.

## 6. Conclusions

SAR, as a new imaging radar system, can perform the high-resolution imaging of large scenes at all times and in all weathers. However, the high-frequency vibration of the airborne platform often restricts imaging accuracy, and the existing SAR imaging algorithms cannot meet the accuracy requirements under vibration conditions. Therefore, this paper focuses on a vibration error model for the arc array SAR system and analyses the impact on imaging when vibration error exists. Theoretically, we deduce the reasons for the appearance of paired echoes when vibration error exists, as well as the influence of vibration amplitude and frequency on paired echoes, and prove the reliability of the theoretical results by combining with simulation analysis. That is, when the vibration error exists, infinite pairs of echoes are generated in the azimuthal direction, and with the increase in vibration frequency, the pairs of echoes are further away from the main image. The size of the vibration amplitude affects the peak value of the main image and the number of echoes; the larger the amplitude is, the smaller the peak value of the main image is, and the greater the number of pairs of echoes is. In addition, this paper proposes a vibration phase estimation and compensation algorithm based on delayed conjugate multiplication, which can effectively eliminate paired echoes, compensate for the vibration phase, and greatly improve the imaging quality. This algorithm is suitable for single-channel systems transmitting linear FM signals. The simulation results show that the proposed method can compensate for the phase error generated by vibration, and the proposed algorithm suppresses false targets without generating new error phases and without widening the main flap of the real target. At the same time, it can effectively solve the vibration compensation problem when there are fewer strong points, and the focusing effect is good.

## Figures and Tables

**Table 1:** Simulation parameters.

Parameter Symbols	Parameter Definition	Parameter Value
*f_c_*	Central Frequency	35.5 GHz
*B_r_*	Signal Bandwidth	1000 MHz
*T_r_*	Sweep Time	0.1 ms
*H*	Platform height	1000 m
*R_arc_*	Arc Array Radius	0.6 m
*θ_a_*	Array Beamwidth (−3 dB)	70°

**Table 2:** Imaging performance analysis.

Target	Range		Azimuth	
	Resolution (m)	PSLR (dB)	Resolution (°)	PSLR (dB)
$P_{1}$	0.616	−13.58	1.354	−12.54
$P_{2}$	0.621	−13.34	1.057	−13.43
$P_{3}$	0.561	−13.31	1.404	−13.29

## Data Availability

Data are contained within the article.
